# The application and mechanism of Chinese medicine in the upstream treatment of atrial fibrillation

**DOI:** 10.3389/fcvm.2023.1229021

**Published:** 2023-08-07

**Authors:** Min Liu, Chenhan Mao, Fusen Zhao, Zhaoyang Chen, Xindong Wang

**Affiliations:** ^1^ The Third Clinical Medical College, Nanjing University of Chinese Medicine, Nanjing, China; ^2^ Jiangsu Province Academy of Traditional Chinese Medicine, Nanjing, China; ^3^ Department of Cardiology, Affiliated Hospital of Integrated Traditional Chinese and Western Medicine, Nanjing University of Chinese Medicine, Nanjing, China

**Keywords:** atrial fibrillation, upstream treatment, Chinese medicine, Chinese herb, review

## Abstract

Upstream treatment of atrial fibrillation (AF, for short) is a new approach to the prevention and treatment of AF with non-antiarrhythmic drugs, which is essentially primary and secondary prevention of AF. The former refers to the prevention of AF by controlling risk factors such as diabetes, hypertension, and heart failure before AF occurs, and the latter mainly refers to targeting ion channels, inflammation, oxidative stress, and other pathways to reduce or reverse atrial electrical and structural remodeling, reduction of AF load, and reduction of the chance of AF occurrence or progression. More and more studies have shown that many traditional Chinese medicines, active ingredients of Chinese medicines, and Chinese herbal formulas have definite effects on the upstream treatment of AF, but their mechanisms of action are different. Therefore, we summarized the relevant literature on the application and mechanisms of Chinese medicine on the upstream treatment of AF in recent years, hoping to be helpful for subsequent studies.

## 1. Introduction

Atrial fibrillation (AF) is a common arrhythmia, which is a serious disorder of atrial electrical activity caused by the loss of regular and orderly atrial electrical activity and its replacement by rapid and disordered fibrillation waves. The high incidence of AF in aging societies, the disabling and fatal nature of thromboembolic complications in stroke, and the chronic, long-lasting, and recurrent nature of the disease indicate an urgent need for noninvasive, convenient, reliable, and safe therapies. However, existing treatments for AF, such as drugs and ablation procedures, are prone to side effects, complications, and high surgical recurrence rates, all with their limitations (1, 2). Currently, we generally pay more attention to the ablation treatment of pulmonary venous sleeve, research found that the pathogenesis of atrial fibrillation is not limited to this, atrial fibrosis, inflammation, ionic currents and channels, oxidative stress, atrial remodeling, RAAS, and autonomic nervous system are closely related to the occurrence and maintenance of AF, but its research is limited. Therefore, we urgently seek ways to intervene in the development of AF at its source.

Upstream treatment is a new approach to prevent and treat AF with non-antiarrhythmic drugs, which is essentially primary and secondary prevention of AF. The former refers to the prevention of AF by controlling risk factors such as diabetes, hypertension, and heart failure before AF occurs, while the latter refers to reducing or reversing atrial electrical and structural remodeling, reducing AF load, and reducing the chance of AF occurrence or progression by targeting ion channels, inflammation, oxidative stress, and so on. Intervene in the occurrence and development of AF from the upstream, will greatly reduce the incidence of AF and improve the quality of patients' life. The prevention and treatment of AF in traditional Chinese medicine (TCM) starts with the patients themselves, using the concept of holism and distinguishing different types of symptoms to identify and treat them, which is not only effective but also can largely reduce or even avoid the toxic and side effects of western drugs. The advantages of natural products in terms of safety and multi-targeting may can be combined with ablation in a complementary way. Therefore it is essential to search for possible effects of natural products on AF and to discover their specific mechanisms.

**Figure 1:**
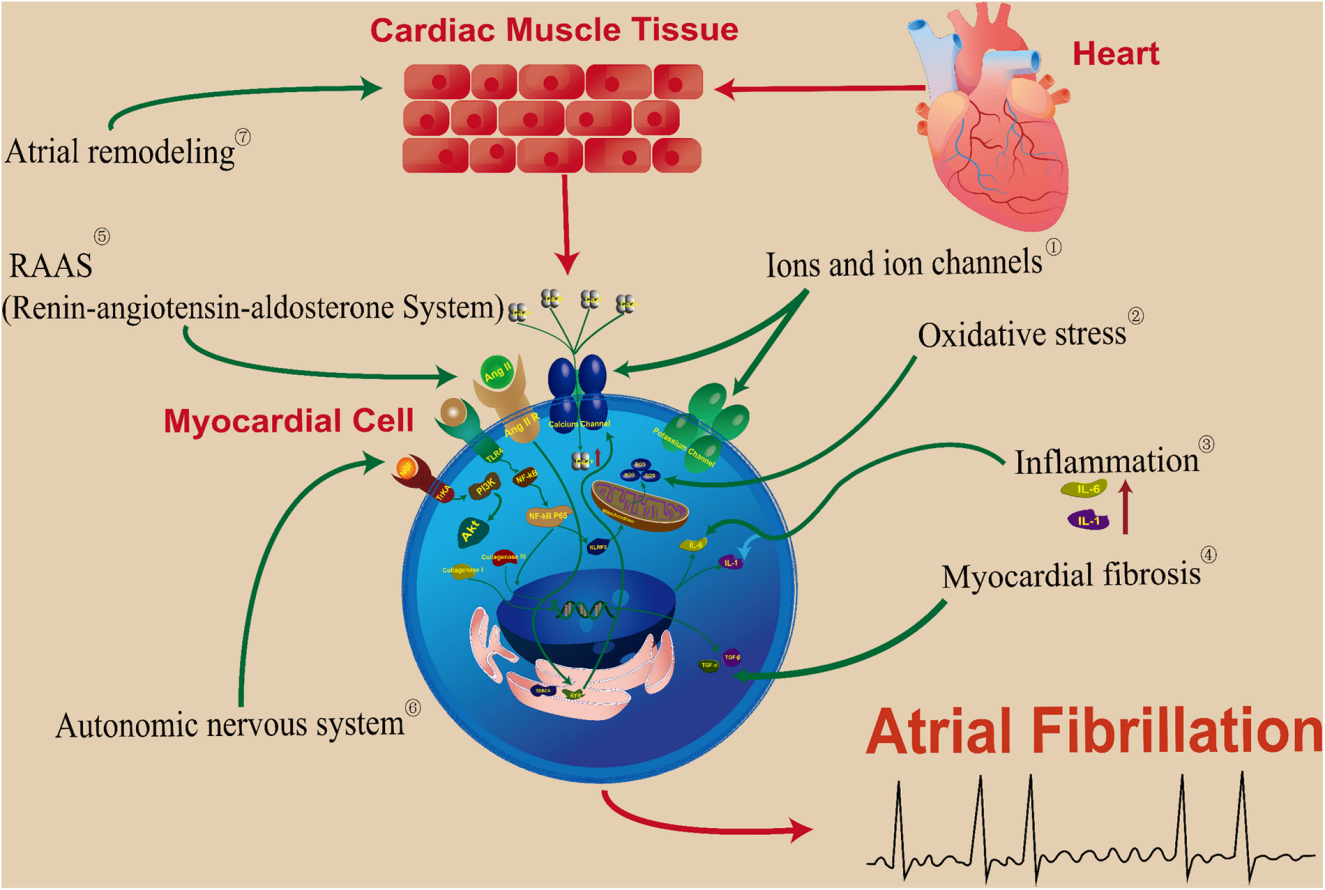
TCM targets for the prevention and treatment of atrial fibrillation upstream. ① Baicalein, Smilax glabra flavonoids, salvianolic acid A, astragalosides, saikosaponin a and d, cyclic vincristine D, resibufgenin. ② Wenxin Keli, Apocynin. ③ Qishen granule, Tongmai Yangxin pill, Xinxuekang capsule, Guizhi Gancao Decoction. ④ Ginseng Dingzhi Decoction, Tongguan capsule, Salvianolic acid B, Rikkunshito. ⑤ Gastrodin, Xin-Jin-Er-Kang. ⑥ Lianxia formula granule. ⑦ Icariin, Danqi soft capsule, Quercetin.

Numerous studies have shown that Chinese medicine has clear advantages for the upstream treatment of AF and is gradually gaining attention, as presented in Figure 1. In this paper, we will review the latest progress on the application and mechanism of upstream treatment of AF.

## 2. Analysis of the entry points of the advantages of Chinese medicine in preventing and treating AF and the current status of research on the treatment method

### 2.1. Analysis of clinical difficulties and advantageous entry points of TCM for AF prevention and treatment

According to its manifestations, AF can belong to the category of “palpitation” in Chinese medicine in mild cases, and “severe palpitations” in more serious cases, which is called “zhengchong” in Chinese. Palpitations are mostly caused by physical weakness, diet, fatigue, excessive emotion, external pathogens, improper medicine and food, etc. Therefore, AF can differentiate its pattern types and susceptibility factors according to its physical constitution to take preventive and curative measures as early as possible, which reflects the “concept of holism” of TCM as well as the “pattern differentiation and treatment”. However, the current situation is that the prevention and treatment of AF in TCM still mostly stay in theoretical research, and the acceptance and recognition of TCM by patients have yet to be improved, so there is a lack of multicenter, large-scale, randomized and parallel high-level clinical trials, which has affected the progress of related research to a certain extent. More importantly, due to the lack of sufficient mature and in-depth studies, we have not yet established mature clinical treatment norms and standards for the prevention and treatment of AF, which also affects the promotion and application of TCM for the prevention and treatment of AF. The results of existing studies have shown that the use of TCM or a combination of TCM and western medicine in the prevention and treatment of AF can improve the electrical and structural remodeling of the atrium, improve atrial fibrosis, control the production of inflammatory factors, and significantly increase the maintenance rate of sinus rhythm in patients, thus significantly improving the quality of survival and safety of patients (3).

### 2.2. Progress of research on treatment method

Modern medical practitioners, have been treating AF with pattern differentiation and treatment, and have achieved good curative effects. According to the TCM pattern types, AF was classified into vigorous fire due to yin deficiency, qi deficiency and blood stasis, deficiency of both qi and yin, and phlegm-heat harassing the heart, and so on. The combination of TCM and antiarrhythmic drugs in the treatment of atrial fibrillation often achieves good results (4). Weiqin Guo (5) believes that the common pathomechanism basis of cardiac disorders is qi deficiency and blood stasis, so treatment emphasizes boosting qi and invigorating blood. Qimei Dong (6) believes that the pathomechanism of paroxysmal AF is “liver wind is the trigger, heart fire is the source, and wind and fire incite each other”, and liver-kidney yin deficiency, internal growth of fire from constraint and deficiency-fire are the sources of AF. Internal wind motivation, wind and fire incite each other, carrying phlegm and stasis, and violating the heart and brain is the key pathomechanism of AF attack and triggering a stroke, so she came up with therapeutic methods that enriching yin and extinguishing wind, draining fire and suppressing palpitations. Weiliang Weng (7) thinks that Yang deficiency and blood stasis are the keys to the occurrence of AF, so the basic treatment is to warm yang and boost qi, invigorate blood and dissolve stasis. It can be seen that most physicians believe that the pathological nature of AF is root deficiency and exterior excess, the former is based on the deficiency of Qi, Yin, and Yang, while the latter is blamed on blood stasis, phlegm and fire, and individual physicians take a different approach and treat “wind” as one of the pathological factors of AF.

## 3. TCM targets for the prevention and treatment of atrial fibrillation upstream

### 3.1. Ions and ion channels

#### 3.1.1. Calcium ions and calcium channels

The L-type Ca^2+^ current (I_Ca-L_) is an important component of the cardiomyocyte action potential and its excitatory function, and abnormal calcium homeostasis is an important mechanism for the occurrence and development of AF (8). Intervening in Ca^2+^ current and calcium ion channels by TCM can provide some new ideas for the prevention and treatment of AF: A study to investigate the possible effects of baicalein on cardiotoxicity in rats showed that, in addition to reducing reactive oxygen species production and inhibiting apoptosis, baicalein also inhibited intracellular calcium ion concentration and reduced calcium overload, all of which reduced cardiac injury (9). The ryanodine receptors are a class of calcium release channels located in the intracellular sarcoplasmic reticulum membrane. It was found that smilax glabra flavonoids have a wide range of anti-cardiac hypertrophic effects by a mechanism related to the inhibition of ryanodine receptors-mediated intracellular calcium release from cardiomyocytes (10). In a subsequent study, it was found that pro-linked protein-2 was also involved in mediating the calcium release response (11). Sarcoplasmic/endoplasmic reticulum calcium ATPase (SERCA) is an important regulatory protein involved in the calcium cycle and is closely related to the occurrence and development of AF. There was an experiment showed that Salvianolic Acid A treatment significantly reversed the effects of arsenic trioxide on SERCA activity in heart tissue, which indicated that it can regulate Ca^2+^-related protein expression levels to prevent calcium overload (12). Moreover, another study proved that Astragalosides can improve left ventricular function and heart structure by reversing the inhibition of SERCA activity and increasing the expression of phospholamban (13). Saikosaponin a and d, the active ingredients of Radix bupleuri, have been found that they are SERCA inhibitors, whose content is reduced and the cardiotoxicity and hepatotoxicity of Radix bupleuri are attenuated after vinegar baking process (14).

#### 3.1.2. Potassium ions and potassium channels

The main physiological functions of the current conducted by potassium channels are to maintain the resting membrane potential, mediate repolarization of the action potential, and respond to changes in intracellular sodium, calcium, and ATP/ADP concentrations. Therefore, potassium homeostasis is essential for the proper functioning of the cardiovascular system. It is now known that the potassium channel encoded by human ether-a-go-go-related gene (hERG) is an important factor in the prolongation of QT interval, which is closely related to the development of AF (15). Cyclovirobuxine D (CVB-D) is the main active ingredient of the commonly used clinical drug, Huangyangning tablets. CVB-D was found to inhibit hERG-encoded potassium channels in concentration-dependence, so it can be speculated that CVB-D can play a role in the upstream treatment of AF by inhibiting hERG (16). Resibufgenin, an extract of the traditional Chinese medicine Chan Su, was found to have a multichannel blocking effect, inhibiting calcium ions and hERG currents in a concentration-dependent manner, significantly prolonging atrioventricular conduction time and slowing down ventricular conduction. However, *in vivo* experiments found that Resibufgenin could induce arrhythmia and even cardiac arrest in guinea pigs at a concentration of 10 μM (17). It can be seen that the effects of TCM on potassium ions and potassium currents are obvious and practical, but relevant studies are still limited, thus the possible effects and mechanisms of TCM on them should be further explored. The action mechanism of the above TCM on ions and ion channels is presented in Table 1.

**Table 1:** TCM preventing and treating upstream AF targets on ions and ion channels.

TCM	Model	Intervention	Cellular and molecular mechanisms	Reference
Baicalein	H9c2	0.5 μM, 1 μM, 5 μM, 10 μM.	Reducing ROS production and inhibiting apoptosis, inhibiting intracellular Ca^2+^ concentration to reduce calcium overload.	(9)
Smilax glabra flavonoids	H9c2	0.25, 0.5, 1.0 mg/ml.	Inhibiting ryanodine receptors-mediated intracellular Ca^2+^ release from cardiomyocytes.	(10, 11)
Salvianolic Acid A	Male BALB/c mice, male SD rats	10 mg/kg/day.	Suppressing Ca^2+^ overload and improving SERCA activity and expression, alleviating calcium homeostasis imbalances, inhibiting endoplasmic reticulum stress by downregulating GRP78, JNK, CHOP and caspase-12.	(12)
Astragalosides	Male Wistar rats	10 ml/kg/d.	Reversing the inhibition of SERCA activity and increasing the expression of phospholamban.	(13)
Saikosaponin a and d	HepG2, H9c2, neonatal rat cardiomyocytes	10 mM.	SERCA inhibitors.	(14)
Cyclic vincristine D	HEK293	1 μM, 5 μM, 10 μM, 20 μM, 30 μM.	Inhibiting hERG-encoded potassium channels in concentration dependence.	(16)
Resibufgenin	Ventricular myocytes, hERG-HEK293	10 μM, 30 μM, 100 μM.	Inhibiting calcium ions and hERG currents in concentration-dependence, prolonging atrioventricular conduction time and slowing down ventricular conduction.	(17)

### 3.2. Inflammation

A large body of evidence shows that AF is closely related to inflammation. Inflammation leads to electrical, structural and autonomic remodeling of AF (18) and involves the development and maintenance of AF. Tumor necrosis factor (TNF-α) is a peptide inflammatory factor that can affect cell growth, differentiation, and the process of apoptosis, and interleukin (IL) is a lymphokine that interacts between leukocytes or immune cells, and IL-2, IL-6, and IL-8 have been found to be associated with inflammation. Some scholars have explored the antagonistic effect of Shenzhu Ningxin Formula on the inflammatory response in AF rats and found that serum TNF-α, IL-1, and IL-6 levels were significantly reduced in AF rats. The current findings suggest that NOD-like receptor protein-3 (NLRP3) is an important predictor of the occurrence and development of AF (19), Qishen granule has been found to exert cardioprotective effects by inhibiting NLRP3 inflammatory vesicles and cellular scorching in rats with myocardial infarction (20). It was found that Tongmai Yangxin pill has significant anti-inflammatory activity, and the mechanism may be related to the regulation of mRNA and protein expression of estrogen receptor 1 and nuclear transcription factor κB (NF-κB) signaling pathway activity (21). Xinxuekang capsule can regulate hypoxia-inducible factor-1α and reduce NF-κB p65 protein expression, alleviate oxidative stress and inflammatory response *in vitro* and *in vivo*, and attenuate adriamycin-induced cardiotoxicity (22). Guizhi Gancao Decoction can reduce myocardial ischemia/reperfusion injury by inhibiting the Toll-like receptor 4/NF-κB signaling pathway to reduce inflammatory response and apoptosis (23). It can be seen that many inflammatory factors are closely related to the development of AF, and the effects of TCM on inflammatory factors are also different, as presented in Table 2, but their specific mechanisms are not yet clear.

**Table 2:** TCM preventing and treating upstream AF targets on inflammation.

TCM	Model	Intervention	Cellular and molecular mechanisms	Reference
Qishen granule	SD rats, H9c2	400 μg/ml, 600 μg/ml, 800 μg/ml, 1,000 μg/ml.	Inhibiting ROS production and decreasing the protein levels of P65-NF-*κ*B, NLRP3, ASC, Caspase-1(P20), Cleaved IL-18, Cleaved IL-1β and NT-GSDMD.	(20)
Tongmai Yangxin pill	RAW264.7	25, 50, 100 μg/ml.	Raising the mRNA and protein expression of estrogen receptor 1, blocking the reduction of IκBα level and the phosphorylation of IKKα/β, IκBα and NF-κB p65, accompanied by inhibiting MCP-1, TNF-α and IL-6 production.	(21)
Xinxuekang capsule	H9c2, 4T1, HepG2, H460	200, 400, 800 ng/ml.	Increasing the protein expression level of hypoxia-inducible factor-1α and decreasing the protein expression level of NF-κB p65.	(22)
Guizhi Gancao Decoction	SD rats	1.8 g/kg, 3.6 g/kg.	Upregulating Bcl-2, PPARα and PPAR*γ*, downregulation of Bax, caspase-3, and caspase-9, attenuating the levels of TNF-α, IL-6, and IL-1β in serum by inhibiting Toll-like receptor 4/NF-κB signaling pathway.	(23)

### 3.3. Oxidative stress

**Figure 2:**
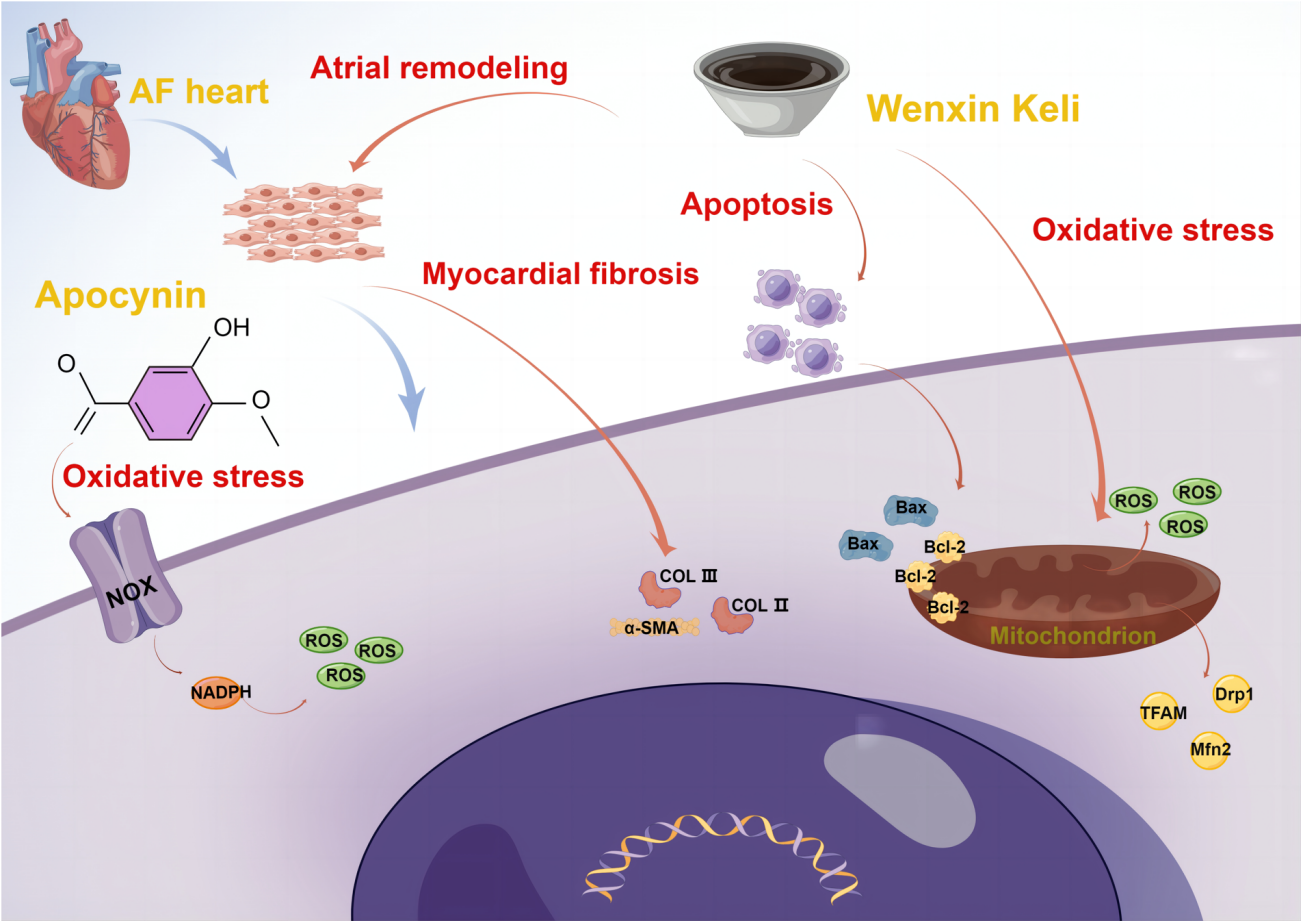
Mechanisms of Wenxin Keli and Apocynin on upstream atrial fibrillation (By Figdraw). AF, atrial fibrillation; Bcl-2, B-cell lymphoma-2; Bax, Bcl2-associated X; ROS, reactive oxide species; TFAM, mitochondrial transcription factor A; Drp1, dynamin-related protein; Mfn2, mitofusin 2; COL II, collagen II; COL III, collagen III; α-SMA, alpha smooth muscle actin; NADPH, nicotinamide adenine dinucleotide phosphate; NOX, NADPH oxidase.

Oxidative stress plays an important role in the development of AF. The accumulation of oxidation products such as reactive oxide species (ROS) predisposes to DNA damage, apoptosis, and necrosis, malondialdehyde (MDA) can indirectly reflect cellular oxidative damage, and superoxide dismutase (SOD) reflects the antioxidant capacity. It was found that Wenxin Keli significantly improved the elevation of ROS level in atrial fibroblasts of Sprague-Dawley (SD) rats induced by hydrogen peroxide, and alleviated the decrease of mitochondrial membrane potential and mitochondrial oxygen consumption. In addition, *in vitro* experiments, it also showed that Wenxin Keli can reduce the serum MDA level, increase the SOD level, and reduce the AF induction rate in diabetic rats (24). It is currently believed that nicotinamide adenine dinucleotide phosphate (NADPH) oxidase is an important source of ROS, and overexpression of NADPH oxidase 2 (NOX2) can mildly induce AF, and apocynin is a non-specific NOX inhibitor that can inhibit the expression of oxidative stress-related protein products. On the one hand, *in vitro* experiments have shown that apocynin has strong free radical scavenging activity and can inhibit oxidative stress levels in experimental rats, thus exerting a protective effect on the heart (25), but on the other hand, the significant arrhythmogenic effects and cardiotoxicity of oleander cannot be ignored (26). It can be seen that TCM can affect many aspects of oxidative stress, thus improving atrial remodeling and reducing the incidence and maintenance of AF, as presented in Table 3 and Figure 2, but its specific effects on the cardiovascular system need more exploration.

**Table 3:** TCM preventing and treating upstream AF targets on oxidative stress.

TCM	Model	Intervention	Cellular and molecular mechanisms	Reference
Wenxin Keli	Fibroblasts, SD rats	1 g/L, 3 g/L; 3 g/kg.	Improving atrial remodeling by regulating mitochondrial function and homeostasis, reducing mitochondrial ROS, reducing apoptosis.	(24)
Apocynin	SD rats	10, 30, 100 mg/kg;	Scavenging free radical, preventing the elevation of marker enzymes such as LDH, GGT, CK-MB, and CPK, AST, ALT, and ALP in plasma, attenuating the lipid peroxidation.	(25)

### 3.4. Myocardial fibrosis

Atrial structural remodeling can lead to irregular conduction of local myocardial electrical activity and the formation of more foldback loops, resulting in blocked conduction within the atrial, which leads to the development and progression of AF. Excessive proliferation and transformation of cardiac fibroblasts, and excessive collagen synthesis are important biological basis of myocardial fibrosis, transforming growth factor (TGF) and other targets are important effective ways against myocardial fibrosis. A study found that Ginseng Dingzhi Decoction can inhibit the expression of collagenase I (COL I) and collagenase III (COL III) in rat cardiomyocytes and reduce the transcript levels of TGF-α and TGF-β (27). Tongguan capsule had been confirmed that it has the same effect of inhibiting the expression of COL I and COL III to improve atrial fibrosis, which also reduced the susceptibility of myocardial infarction rats to AF (28). Some scholars found that after being treated with Salvianolic Acid B, the degree of atrial fibrosis was significantly reduced, its mechanism may relate to that Salvianolic Acid B can reduce the stimulating effects of Ang II on the accumulation of extracellular matrix via miR-148a-3p induction by activating AMP-activated protein kinase (AMPK)/forkhead box proteins O1 (FOXO1) pathway (29). Rikkunshito, named “Liu-Jun-Zi Decoction” in Chinese, is a traditional herbal medicine widely used in patients with gastrointestinal symptoms, it was found that Rikkunshito prevented atrial fibrosis and attenuated enhanced vulnerability to AF induced by angiotensin II, the mechanism was related to the enhancement of growth hormone secretagogue receptor-silent information regulator 1 (SIRT1) pathway (30). Obviously, TCM can inhibit atrial fibrosis, protect cardiomyocytes and improve cardiac function through multi-target pathways (Table 4).

**Table 4:** TCM preventing and treating upstream AF targets on myocardial fibrosis.

TCM	Model	Intervention	Cellular and molecular mechanisms	Reference
Ginseng Dingzhi Decoction	C57BL/6J mice, C57 mice, Mouse cardiomyocytes.	32 g/kg/d.	Inhibiting the expression of collagenase I and collagenase III and reducing the transcript levels of TGF-α and TGF-β.	(27)
Tongguan capsule	SD rats	0.6, 1.2 g/kg/day.	Inhibiting the expression of collagenase I and collagenase III by inhibiting the proliferation, migration, differentiation and cytokine secretion of cardiac fibroblasts.	(28)
Salvianolic acid B	Male BALB/c mice	200 mg/kg/day.	Reducing the stimulating effects of Ang II on the accumulation of extracellular matrix *via* miR-148a-3p induction by activating AMPK/FoxO1 pathway.	(29)
Rikkunshito	Male wild-type C57BL/6 mice	1,000 mg/kg/day.	Attenuating the Ang II-induced suppression of sirtuin 1 translocation to the nuclei, suppressing Ang II-induced phosphorylation of IκB, overexpression of p53, cellular apoptotic signals and apoptosis.	(30)

### 3.5. Autonomic nervous system

When autonomic nerves (including sympathetic and parasympathetic nerves) are stimulated, the corresponding neurotransmitters are released to bind to receptors, which can induce AF by affecting the permeability of various ion channels in the myocardial cell membrane. In addition, high catecholamine levels after sympathetic activation and parasympathetic nerve stimulation can lead to the shortening of cardiac effective refractory period (ERP) and subsequently induce AF occurrence (31, 32). In addition, the interaction of sympathetic and parasympathetic inhibition or activation of the heart is also an important factor in the development of AF (33). The nerve growth factor (NGF)/tyrosine kinase receptor A (TrKA)/phosphatidylinositol 3-kinase (PI3K)/protein kinase B (Akt) signaling pathway is an important target for reducing sympathetic remodeling after myocardial infarction (34, 35). Some scholars found that Lianxia Formula Granule could inhibit the expression of key proteins and mRNAs of NGF/TrKA/PI3K/AKT signaling pathway and suppress catecholamine levels, thus attenuating sympathetic remodeling and reducing arrhythmia susceptibility (36), as presented in Table 5. Current research on the effects of TCM on the autonomic nervous system of AF is relatively limited, and more exploration is needed.

**Table 5:** TCM preventing and treating upstream AF targets on autonomic nervous system.

TCM	Model	Intervention	Cellular and molecular mechanisms	Reference
Lianxia Formula Granule	SD rats	189.00, 94.50, 47.25 mg/kg/d.	Inhibiting the expression of key proteins and mRNAs of NGF/TrKA/PI3K/AKT signaling pathway, suppressing catecholamine levels, thus attenuating sympathetic remodeling.	(36)

### 3.6. RAAS (renin-angiotensin-aldosterone system)

The RAAS is involved in the occurrence of AF, and its mechanism may be related to the electrical and structural remodeling of the atria (37). It has been found that there are different degrees of RAAS activation in AF, and angiotensin II (Ang II) can cause not only intracellular calcium overload but also transient outward potassium current, ion channel protein expression, and current density abnormalities, angiotensin-converting enzyme inhibitors (ACEI) and Ang II receptor blockers (ARB) can inhibit the atrial ERP shortening caused by this response. The results of established studies have shown that numerous TCMs have an inhibitory effect on the RAAS on the cardiovascular system (Table 6). It was found that gastrodin could reduce blood pressure in spontaneously hypertensive rats, and its mechanism may be related to the direct reduction of Ang Ⅱ level or indirect inhibition of the RAAS by activating peroxisome proliferator-activated receptor (PPARγ) (38). Xin-Jin-Er-Kang was verified that it can improve blood pressure, and cardiovascular and renal function in rats with hypertensive heart failure model, inhibited the activation of RAAS induced by a high salt diet, enhanced the viability of cardiomyocytes by inhibiting the activity and expression of calpain (39). The anti-heart failure and hypotensive effects of many drugs targeting the RAAS have been confirmed experimentally, but the evidence for secondary prevention of AF has not been fully explored, so exploring the effective effects of TCM on the RAAS is of great importance for the upstream treatment of AF.

**Table 6:** TCM preventing and treating upstream AF targets on RAAS.

TCM	Model	Intervention	Cellular and molecular mechanisms	Reference
Gastrodin	SH rats, Wistar-Kyoto rats	4 mg/kg/d.	Reducing Ang Ⅱ level or inhibiting the RAAS by activating PPARγ.	(38)
Xin-Jin-Er-Kang	KM mice, Male Kunming mice	2, 8, 12 g/kg/day.	Inhibiting the activation of RAAS, enhancing the viability of Ang II-attacked cardiomyocytes by inhibiting the activity and expression of calpain.	(39)

### 3.7. Atrial remodeling

Atrial remodeling mainly includes electrical remodeling and structural remodeling of atrial, it refers to the structural and functional changes of atrial myocytes in response to the internal or external stimuli, and is one of the basic triggers of AF (40). The main manifestations of atrial remodeling are ventricular fibrosis, myocardial cell degeneration, mitochondrial enlargement and sarcoplasmic reticulum rupture (41). A large amount of evidence shows that traditional Chinese medicine can play a cardioprotective role by improving atrial remodeling (Table 7): Icariin is the main active component of Chinese herb Epimedium, which has been demonstrated to exert potential antiarrhythmic effect. Icariin can improve the electrical and structural remodeling induced by excessive alcohol consumption, and reduce the induction rate of AF, its mechanism may be related to the targeting of SIRT3-AMPK signal and the protection of mitochondrial dynamics (42). Danqi soft capsule is a Chinese herb medicine, one study investigated its role in left atrial remodeling and atrial fibrillation in rats with heart failure induced by myocardial infarction, finding that Danqi soft capsule meliorates left atrial remodeling by inhibiting the cardiac fibroblasts' function of proliferation, migration, collagen secretion and myofibroblast differentiation, thereby reducing the risk of AF (43). It was also found that Quercetin can effectively inhibit the expression of miR-223-3p in AF model cells and rat myocardial tissue, enhance the expression of FOXO3, activate autophagy pathway, significantly inhibit myocardial fibrosis, and improve myocardial remodeling in AF (44).

**Table 7:** TCM preventing and treating upstream AF targets on atrial remodeling.

TCM	Model	Intervention	Cellular and molecular mechanisms	Reference
Icariin	Male C57BL/6J mice	50 mg/kg/day.	Improving atrial mitochondrial dynamics and repressing mitochondrial ROS damage by re-activating atrial SIRT3-AMPK signaling.	(42)
Danqi soft capsule	Male SD rats	0.6 g/kg, 1.2 g/kg.	Decreasing fibrosis and increasing Cx43 expression, inhibiting fibrosis by modulating the functions (inhibiting proliferation, migration, collagen secretion, and myofibroblasts differentiation) in cardiac fibroblasts.	(43)
Quercetin	Human embryonic kidney cell 293 T, Primary rat cardiac fibroblasts, Wistar rats.	3 mg/kg/day.	Inhibiting the expression of miR-223-3p in AF model cells and rat myocardial tissue, enhancing the expression of FOXO3, activating autophagy pathway, inhibiting myocardial fibrosis.	(44)

## 4. Chinese herbal medicines with preventive and curative effects on upstream atrial fibrillation and potential evidence

### 4.1. Classic prescriptions and empirical compound prescriptions

Zhigancao decoction, a classic formula for the treatment of various types of arrhythmias in Chinese medicine, can effectively improve the clinical symptoms of patients and reduce the incidence of adverse reactions. Zhigancao decoction significantly reduced MMP-9 (matrix metalloproteinase-9) protein expression in AF rabbits and attenuated myocardial fibrosis, shortened field action potential duration (45). In addition, the whole-cell membrane clamp technique was used to observe the pharmacological mechanism of Zhigancao decoction affecting the electrical and structural remodeling of rabbit atria to prevent AF, which showed that the I_Ca−L_ action period duration (APD) of rabbit atrial myocytes in the Zhigancao decoction group was significantly prolonged and the atrial ERP was prolonged too (46). All these responses could exert upstream therapeutic effects on AF.

Dingji Fumai Decoction is a classic Chinese medicine compound with good anti-arrhythmic effect and high safety, but its effective components and specific mechanism of anti-atrial fibrillation are still unclear. Some scholars have explored this, they found that Dingji Fumai Decoction significantly increased the level of SIRT1 and decreased the level of ACE, vascular cell adhesion molecule-1 (VCAM-1), and IL-6 in mouse cardiomyocytes, suggesting that the anti-AF effect of Dingji Fumai Decoction can be achieved by regulating cardiac oxidative stress and reducing inflammation (47). Connexin 43 (Cx43) is the most predominantly expressed protein in the myocardium, and the results of a study showed that Dingji Fumai Decoction inhibited potassium current in a dose-dependent manner, and inhibited the activation of NA-K-ATPase and Cx43 at the same time, suggesting that Dingji Fumai Decoction may also have certain effects on ion currents and ion channels (48).

Jianxin Pinglu pill has the effects of boosting qi and nourishing the heart, resolving phlegm and invigorating blood, which is commonly used in the treatment of cardiovascular system diseases such as arrhythmia and ischemic cardiomyopathy. It was found that Jianxin Pinglu pill could prevent ischemia/reperfusion injury-induced arrhythmias in rats, and the mechanism may be related to the upregulation of water channel protein 4 expression level in myocardium and reduction of intracellular edema (49). The mechanism of above Chinese medicinal formulae was demonstrated in Table 8.

**Table 8:** Chinese medicinal formulae for a preventive and curative effect on upstream AF.

Chinese medicinal formula	Model	Invention	Cellular and molecular mechanisms	Reference
Zhigancao decoction	New Zealand rabbits	20 g/kg/d.	Reducing matrix MMP-9 protein expression and attenuating myocardial fibrosis.	(45)
	New Zealand rabbits	25.95 g/ml.	Prolonging the I_Ca-L_ APD and atrial ERP.	(46)
Dingji Fumai Decoction	HL-1 cells	1.0–1.1 g/L, 0.25–1.0 g/L, 1.0 g/L.	Increasing the level of SIRT1 and decreasing the level of ACE, VCAM-1, and IL-6.	(47)
	SD rats	17.6 g/kg.	Inhibiting potassium current in a dose-dependent manner, and inhibiting the activation of NA-K-ATPase and Cx43 at the same time.	(48)
Jianxin Pinglu pill	SD rats	3.945 g.	Upregulating water channel protein 4 expression level in myocardium and reducting intracellular edema.	(49)

### 4.2. Chinese herbs

Arnebiae Radix is good at clearing heat and cooling the blood, invigorating blood and resolving toxins, promoting eruption of papules and dissolving macules, and contains a variety of chemical components such as naphthoquinones, polysaccharides, monoterpene phenols and benzoquinones, and esters, which has a variety of pharmacological activities such as anti-inflammatory, anti-tumor, antibacterial, hepatoprotective and immunomodulatory (50). Modern studies have shown that treatment with Arnebiae Radix liquor in AF models reduced the induction, duration, and susceptibility to AF in rats with AF. In addition, Arnebiae Radix significantly reduced the degree of atrial fibrosis and inhibited acetylcholine-calcium chloride-induced atrial enlargement, thus improving atrial remodeling, and rats' cardiac function was also significantly improved (51).

Coptidis rhizoma has the effect of clearing heat and drying dampness, draining fire and resolving toxins, modern doctors often add Coptidis rhizoma to clear the heart and fire for phlegm-heat disturbing the heart type AF patients, often with good results. Modern pharmacological researches show that Coptidis rhizoma has anti-arrhythmic, anti-heart failure, myocardial protection, hypotensive, hypoglycemic, anti-inflammatory, and other effects (52). It can play a preventive role against AF through the effective management of hypertension, diabetes and its cardiovascular complications, heart failure, and other diseases. Some scholars have analyzed the relationship between the active components of Coptidis rhizoma and AF targets by network pharmacology methods, and the results showed that Coptidis rhizoma can inhibit gene expression, inhibit megakaryocyte differentiation, and anti-protein metabolism, which together exerted anti-AF effects (53).

Panax notoginseng has anticoagulant, antithrombotic, anti-inflammatory, antioxidant, and other effects, contains saponins, polysaccharides, flavonoids, alkynes, alcohols, and other chemical components, its main medicinal components are saponin compounds, panaxanthin, protein and amino acid components (54). The main medicinal components are saponins, dencichine, proteins, and amino acids. Experiments have demonstrated that Panax notoginseng can effectively reduce myocardial fibrosis, IL-6, albumin, AKT1, TNF and vascular endothelial growth factor A (VEGFA) are the five most critical action targets, and the advanced glycosylation end-product-advanced glycosylation end-product receptor (AGE-RAGE) signaling pathway is the most important potential pathway for Panax notoginseng to treat myocardial fibrosis in diabetic complications (55). Some studies have shown that Panax notoginseng has no significant effect on controlling the ventricular rate of AF but can reduce the incidence of thromboembolic events, and the combination of Chinese and Western medicine in the treatment of AF can reduce the dose of anticoagulant drugs and reduce side effects, and to a certain extent can prevent the occurrence of stroke, which has a more significant safety, reflecting the advantages of combining Chinese and Western medicine (56).

Ginseng, as a herb with high medicinal value, has pharmacological effects such as anti-fatigue, anti-aging, anti-oxidation, and immunity enhancement, and contains various components such as ginsenosides, ginseng polysaccharides, volatile oils (terpenoids, alcohols, fatty acids, etc.) and amino acids (57). Some scholars have investigated the protective effects of ginseng in ischemia/reperfusion injury and found that ginseng preparations can increase serum nitric oxide production, reduce the serum activity of creatine kinase and lactate dehydrogenase (LDH), significantly reduce the infarct size and decrease the incidence of arrhythmia (58). In addition, ginseng has been found to have therapeutic effects in type 2 diabetes, and its core targets include insulin resistance, hypoxia-inducible factor 1 signaling pathway, PI3K/AKT signaling pathway, and prolactin signaling pathway, etc (59). We also listed the specific mechanism of Chinese herbs on upstream AF in Table 9.

**Table 9:** Chinese herbs for a preventive and curative effect on upstream AF.

Chinese herbs	Model	Intervention	Cellular and molecular mechanisms	Reference
Arnebiae Radix	SD rats	0.18 g/ml.	Reducing the degree of atrial fibrosis and inhibiting CaCl_2_-Ach-induced atrial enlargement, thus improving atrial remodeling, and improving rats’ cardiac function, too.	(51)
Ginseng	SD rats	Ginsenoside Re (10 mg/kg), Rb1 (10 mg/kg), Rg1 (10 mg/kg), or a mixture (10 mg/kg).	Increasing serum NO production, reducing the serum activity of creatine kinase and LDH, significantly reducing the infarct size and decreasing the incidence of arrhythmia.	(58)

### 4.3. Preparation of Chinese medicine

Wenxin Keli is based on Zhigancao Decoction, with the effect of boosting qi and nourishing yin, invigorating blood and dissolving stasis, which is clinically effective and safe for arrhythmias of various causes (60). It was found that Wenxin Keli can selectively inhibit sodium channel currents and have a significant inhibitory effect on acetylcholine (Ach)-induced AF (61). Ganglionic plexi ablation is a safe and efficacious method to improve pulmonary vein isolation in patients with AF (62), some scholars investigated the effect of Wenxin Keli on recurrent AF and atrial matrix remodeling after epicardial ganglionic plexi ablation, finding that Wenxin Keli can reduce the incidence of postoperative AF and improve atrial matrix remodeling, including Cx43 upregulation and increased levels of atrial natriuretic peptide, TNF-α and IL-6 (63). It can be seen that Wenxin Kili has definite upstream therapeutic effects on AF in many ways.

Shensong Yangxin capsule is a traditional Chinese medicine that has been used widely to treat arrhythmia. Shensong Yangxin capsule was found to down-regulate MMP-9 and MMP inhibitor levels and improve left atrial conduction function by inhibiting left atrial fibrosis, which contributes to prevent the development of a myocardial infarction induced vulnerable substrate for AF (64). Shensong Yangxin capsule can also reduce metabolic syndrome-induced AF sensitivity by upregulating iron transporter protein expression to reduce iron overload, thereby inhibiting cardiac electrical and structural remodeling (65). Some scholars found that Shensong Yangxin capsule significantly inhibited sympathetic enhancement in dogs, suppressed the decrease of Ach and *α*7 nicotinic acetylcholine receptor protein induced by long-term intermittent atrial pacing, and hindered the progression of atrial electrical remodeling and AF (66).

Huangyangning is a Chinese medicinal preparation made from CVB-D extracted from the plant boxwood, which is good at moving qi and invigorating blood, unblocking the collaterals and relieving pain, and is commonly used in coronary heart disease and arrhythmia caused by qi stagnation and blood stasis. In addition to the above-mentioned inhibition of hERG-encoded potassium channels (16), CVB-D inhibited I_Ca-L_ in a concentration-dependent manner and increased caffeine-induced Ca^2+^ release in ventricular myocytes, and Yu et al. showed that CVB-D can promote calcium utilization while preventing calcium loss (67). In terms of oxidative stress, CVB-D was found to inhibit oxidative stress by activating the Nrf2 signaling pathway, inhibit cardiomyocyte scorching by suppressing NLRP3 expression, and improve cardiac function and survival in rats with diabetic cardiomyopathy (68, 69), protect rat aortic endothelial cells from hypoxia and enhance NO release from endothelial cells (70), alleviate adriamycin-induced myocardial oxidative damage (which is mainly manifested by lipid peroxidation and protein carbonylation and reduced ratio of glutathione (GSH) to oxidized glutathione (GSSG). In addition, CVB-D has been found to prevent mitochondrial damage (71). CVB-D may induce arrhythmias by a mechanism that may be related to the excessive prolongation of APD and the inhibition of resting potential, action potential amplitude and maximum depolarization rate (72). Therefore, this drug should be used with caution in clinical application. The above preparation of Chinese medicine's mechanism was demonstrated in Table 10.

**Table 10:** Preparation of Chinese medicine for a preventive and curative effect on upstream AF.

Preparation of Chinese medicine	Model	Intervention	Cellular and molecular mechanisms	Reference
Wenxin Kili	HEK5 cells, dogs	30 g/L.	Inhibiting sodium channel currents and prolonging ADP.	(61)
	Dogs	0.25 g/kg/day.	Inhibiting atrial Cx43 remodeling and significantly attenuating the levels of ANP, TNF-α and IL-6.	(63)
Shensong Yangxin capsule	SD rats	600 mg/kg/d.	Decreasing left atrial fibrosis, downregulating TGF-β1, MMP-9, MMP inhibitor, and COL I and COL III expressions, and inhibiting the differentiation of cardiac fibroblasts to myofibroblasts.	(64)
	Wistar rats	0.4, 0.8 g/kg.	Up-regulating Fpn, inhibiting electrical remodeling and structural remodeling, decreasing intracellular iron overload, and reducing ROS production.	(65)
	Adult mongrels	Unavailable	Inhibiting sympathetic enhancement, suppressing the decrease of Ach and α7 nicotinic acetylcholine receptor protein, and hindering the progression of atrial electrical remodeling and AF.	(66)
Huangyangning	SD rats	0.1, 0.5, 1.0, 2.0, 10 μM.	Increasing the release and uptake of Ca^2+^ in systolic and diastolic period, respectively, not only facilitating the utilization of intracellular Ca^2+^, but also preventing the loss of Ca^2+^.	(67)
	SD rats	1, 2 mg/kg/day, 5 mg/kg/2 week.	Activating the Nrf2 signalling pathway to suppress oxidative stress.	(68)
	C57BL/6 mice, primary neonatal rat cardiomyocytes	0.5 mg/kg/day.	Inhibiting NLRP3 expression, increasing cell viability, attenuating cytopathological changes and inhibiting the expression levels of pyroptosis-related proteins.	(69)
	C57BL/6 mice	4 mg/kg/d.	Alleviate myocardial oxidative damage(which is mainly manifested by lipid peroxidation and protein carbonylation and reduced ratio of GSH to GSSG.), preventing mitochondrial damage.	(71)
	SD rats	13.3–63.3 μM/L	Prolonging APD and ERP of ventricular muscle as well as increasing the ERP/APD ratio.	(72)

### 4.4. Active ingredients of Chinese medicine

Salvianolic acid (salvianolic acid A and salvianolic acid B) is an active ingredient extracted from the Chinese medicine Salvia miltiorrhiza (Danshen), which has effects on oxidative stress, myocardial fibrosis, platelet aggregation, coagulation, thrombosis, endothelial dysfunction and inflammation in the cardiovascular system (73), together to play a protective role in cardiovascular diseases. It was found that salvianolic acid B inhibited TGF-*β*1/Smad2/3-mediated collagen deposition and suppressed the thioredoxin-interacting protein (TXNIP)/NLRP3/IL-1β and IL-18 signaling pathways, significantly improving cardiac function and reducing susceptibility to AF and duration (74).

Tanshinone IIA is a fat-soluble active ingredient of salvia miltiorrhiza, with anticoagulant, anti-inflammatory, antioxidant, anti-fibrotic, and immunomodulatory effects (75). The potential electrophysiological mechanism of the antiarrhythmic effect of Tanshinone IIA in rats with chronic heart failure was investigated, and it was found that Tanshinone IIA prolonged the post-atrial repolarization period and interventricular conduction time, and was shown to be effective in reducing the inducibility of AF (76). Differentiation of atrial fibroblasts into myofibroblasts plays a key role in atrial fibrosis. A study suggested that Tanshinone IIA decreased alpha smooth muscle actin (α-SMA), Coll I and Coll III expression, significantly inhibited ROS production and TGF-β1 expression, blocked Ang II-induced the differentiation of atrial fibroblasts to myofibroblasts partly at least (77).

Panax notoginseng saponins (PTS) is the main active component of the Chinese medicine Panax notoginseng, which is often used clinically in the treatment of myocardial ischemia, atherosclerosis, coronary artery disease, and myocardial fibrosis. A study found that PTS had an exertremarked antiarrhythmic activity on coronary artery ligation induced ischemic and reperfused arrythmias in rats, and also produced a significant protective effect on CaCl_2_-Ach induced atrial fibrillation and/or flutter in mice, its mechanism may be produced by antagonizing calcium (78).

Curcumin is an acidic polyphenol extracted from the rhizome of turmeric, etc. It has anti-inflammatory, anti-oxidative stress, anti-fibrotic, immunomodulation, antibacterial, anti-ischemic, and other pharmacological effects (79). An experiment found that plasma levels of IL-17A, IL-1β, IL-6, and TGF-β1 were significantly reduced in the curcumin-treated group, and curcumin significantly shortened the duration of AF and significantly inhibited left atrial fibrosis, its bioinformatics analysis indicated that the IL-17 signaling pathway was the key to curcumin treatment of AF (80). However, another study investigated the effect of nanocurcumin on the incidence of AF, and markers of inflammation and oxidative stress level after coronary artery bypass graft surgery, finding that curcumin treatment did not significantly improve the incidence of AF, and the levels of C-reactive protein (CRP), MDA and GSH levels were not significantly changed evidently (81). Perhaps the role of curcumin in AF needs to be further explored.

Resveratrol is a natural polyphenol, mainly from grapes, peanuts, Polygonum cuspidatum, etc, which has anti-cancer, anti-infection, cardiovascular protection, hepatoprotection, anti-platelet aggregation, and other effects (82). An experiment observed the acute electrophysiologic effects of polyphenols resveratrol and piceatannol in rabbit atria, finding that they increased the atrial refractory period and slow the conduction, and may be further developed as a potential drug for AF (83). Zhong et al. evaluated the therapeutic effect of resveratrol in reducing the occurrence of AF in a heart failure model and explored the underlying mechanisms, finding that myocardial fibrosis was significantly reduced in the resveratrol group, ion channels including Kv1.4, Kv1.5, KvLQT1, Kir2.1, and Nav1.5 were significantly upregulated, and PI3K, AKT, and eNOS mRNA and protein expression were also significantly enhanced (84). Current findings suggest that rheumatoid arthritis can cause atrial remodeling and induce the development and maintenance of AF (85). Accordingly, some scholars found that resveratrol can reverse rheumatoid arthritis-induced atrial structural and metabolic remodeling, then slow down the development of AF (86).

Matrine, an alkaloid extracted from the Chinese herb Sophora flavescens Ait (Kushen), is effective in preventing and improving chronic diseases such as cardiovascular diseases and tumors (87). Matrine can down-regulate M3 receptor-activated delayed rectifier K^+^ current (I_KM3_) density as well as M3 receptor expression, up-regulate I_Ca-L_ density and *α*1C/Cav1.2 expression, reduce AF incidence, and decrease AF duration (88). Matrine also has a significant role in the inhibition of myocardial fibrosis: matrine may reduce AF susceptibility after myocardial infarction by inhibiting the proliferation, migration, differentiation, and secretory capacity of fibroblasts (89).

Berberine is a quaternary ammonium alkaloid isolated from the Chinese medicine Rhizoma Coptidis, which has a wide range of cardioprotective, anti-atherosclerotic, lipid-lowering, anti-obesity, and anti-hepatic steatosis effects on the cardiovascular system (90). Clinical observations have shown that the incidence of AF after coronary artery bypass grafting was significantly reduced after oral administration of berberine, with a significant reduction in serum lipopolysaccharide, CRP, and IL-6 levels (91). Berberine could inhibit the occurrence of Ach-induced AF in rabbits by increasing atrial ERP and prolonging the APD of atrial myocytes (92). Berberine was also found to be an inhibitor of hERG and KCNQ1/KCNE1, but the blocking effect on the latter two was significantly lower than that of hERG (93). Table 11 showed the TCM-based bioactive compounds mechanisms on AF upstream targets.

**Table 11:** TCM-based bioactive compounds and AF upstream targets.

Bioactive compounds	Model	Intervention	Cellular and molecular mechanisms	References
Salvianolic acid	SD rats	10, 20 and 40 mg/kg/day.	Inhibiting TGF-β1/Smad2/3-mediated collagen deposition and suppressing the thioredoxin-interacting protein/NLRP3/IL-1β and IL-18 signaling pathways.	(74)
Tanshinone IIA	Rabbits	10 μm.	Prolonging the post-atrial repolarization period and interventricular conduction time, decreasing α-SMA, Coll I and Coll III expression.	(76, 77)
	Human adult atrial fibroblasts	0, 5, 25, 50, 100, 200 μM	Inhibiting ROS production and TGF-β1 expression, blocking the differentiation of atrial fibroblasts to myofibroblasts.	(77)
Panax notoginseng saponins	Wistar rats	30, 62.5, 100, 300, 500 mg/kg.	Inhibiting sympathetic efferent activity, prolonging APD and ERP, reducing myocardial automaticity, and antagonizing calcium.	(78)
Curcumin	SD rats	112 mg/kg/day.	Reducing the plasma levels of IL-17A, IL-1β, IL-6, and TGF-β1, shortening the duration of AF and significantly inhibiting left atrial fibrosis.	(80, 81)
Resveratrol	Female rabbits	50 µM.	Increasing the atrial refractory period and slowing the conduction.	(83)
	Rats	2.5 mg/kg.	Reducing myocardial fibrosis, upregulating ion channels including Kv1.4, Kv1.5, KvLQT1, Kir2.1, and Nav1.5, and enhancing PI3K, AKT, and eNOS mRNA and protein expression.	(84)
	Wistar rats	10 mg/kg/day.	Reversing atrial structural and metabolic remodeling, preventing and treating MS by mechanisms involving SIRT1, AMPK, and the RAAS.	(86)
Matrine	Mice	15, 30, 45 mg/kg/day.	Down-regulating I_KM3_ density as well as M3 receptor expression, up-regulating I_Ca−L_ density and α1C/Cav1.2 expression, blocking hERG channel.	(88)
	SD rats	50, 100 mg/kg/d.	Inhibiting the proliferation, migration, differentiation, and secretory capacity of fibroblasts, inhibiting the expression levels of inflammatory factors, reducing ROS production, lowering MDA, TGF-β levels, and the activities of PPARβ and PPARγ1, inhibiting the expression of PERK signaling pathway.	(89)
Berberine	Patients	1.2 g/day.	Reducing serum lipopolysaccharide, CRP, and IL-6 levels, alleviating the levels of intestinal endotoxin and systemic inflammation.	(91)
	Adult New Zealand white rabbits	1 or 2 mg/kg Ber.	Increasing atrial ERP and prolonging the APD of atrial myocytes.	(92)
	XenopusOocytes, HEK-293Cells	0.01, 0.1, 1, 10, 100 μM.	Inhibiting hERG and increasing APD, blocking KCNQ1/KCNE1currents.	(93)

## 5. Discussion

In this review, we retrospectively analyzed the application and mechanism of TCM in the upstream treatment of AF. Research on the upstream treatment of AF in TCM is currently focused on atrial myofibrosis, oxidative stress, inflammatory response, atrial remodeling, and ionic currents and channels, while research on the possible role and mechanisms of the autonomic nervous system and the RAAS is insufficient. In addition to the influence on specific mechanisms, the influence of TCM on risk factors related to AF also needs to be further explored, such as hypertension, heart failure, heart valvular disease, sleep apnea, obesity, excessive drinking, etc. The non-pharmaceutical therapies of TCM, including acupuncture, massage, auricular plaster therapy, etc, may also have a role in the upstream of AF. Due to TCM lacks a more in-depth understanding of the etiology and pathogenesis of AF, the treatment methods and prescriptions are not rigorous enough, and there is also a lack of clear mechanism targets and relevant clinical efficacy data support, hence the participation rate and recognition in the prevention and treatment of AF is on the low side. Therefore, it is an urgent and meaningful task to conduct research on the etiology, treatment, and prescription of AF based on traditional Chinese medicine theory, and to explore and discover Chinese medicines that have therapeutic effects on the upstream factors of AF and thus exert anti-AF effects, and to elucidate their targets and mechanisms.

Anyway, despite suffering from lack of sufficient mechanisms and clinical studies, the upstream research of TCM on AF is enough to surprise people, which may provide more drugs.
